# CD27-IgD- memory B cells are modulated by *in vivo* interleukin-6 receptor (IL-6R) blockade in rheumatoid arthritis

**DOI:** 10.1186/s13075-015-0580-y

**Published:** 2015-03-14

**Authors:** Zafar Mahmood, Khalid Muhammad, Marc Schmalzing, Petra Roll, Thomas Dörner, Hans-Peter Tony

**Affiliations:** Department of Medicine II, Rheumatology and Clinical Immunology, University of Würzburg, Oberdürrbacher Str. 6, D-97080 Würzburg, Germany; Department of Molecular Pathology, Institute of Pathology University of Würzburg, Josef-Schneider-Str. 2, D-97080 Würzburg, Germany; CC12, Department of Medicine /Rheumatology and Clinical Immunology, Charite Universitätsmedizin Berlin and DRFZ Berlin, Charitéplatz 1, D-10117 Berlin, Germany

## Abstract

**Introduction:**

Enhanced B cell activity, particularly memory B cells have gained interest in evaluating response during therapies with biologics. CD27-IgD- double-negative (DN) B cells lacking the conventional memory marker CD27 are reported to be part of the memory compartment, however, only scarce data is available for rheumatoid arthritis (RA). We therefore focused on DN B cells in RA, studied their isotypes and modulation during interleukin-6 receptor (IL-6R) inhibition by tocilizumab (TCZ).

**Methods:**

DN B cells were phenotypically analyzed from 40 RA patients during TCZ at baseline week 12, week 24 and 1 year. A single B cell polymerase chain reaction (PCR) approach was used to study Ig receptors, V_H_ gene rearrangements and specific isotypes.

**Results:**

Phenotypic analysis showed a significantly expanded population of DN B cells in RA which contain a heterogeneous mixture of IgG-, IgA- and IgM-expressing cells with a clear dominance of IgG+ cells. DN B cells carry rearranged heavy chain gene sequences with a diversified mutational pattern consistent with memory B cells. In contrast to tumor necrosis factor alpha (TNF-α) inhibition, a significant reduction in mutational frequency of BCR gene rearrangements at week 12, 24 and 1 year (*P* <0.0001) was observed by *in vivo* IL-6R inhibition. These changes were observed for all BCR isotypes IgG, IgA and IgM at week 12, 24 and 1 year (*P* <0.0001). IgA-RF, IgA serum level and IgA+ DN B cells decreased significantly (*P* <0.05) at week 12 and week 24 during TCZ. Patients with a good European League Against Rheumatism (EULAR) response to TCZ had less DN B cells at baseline as compared to moderate responders (*P* = 0.006). Univariate logistic regression analysis revealed that the frequency of DN B cells at baseline is inversely correlated to a subsequent good EULAR response (*P* = 0.024) with an odds ratio of 1.48 (95% confidence interval as 1.05 to 2.06).

**Conclusions:**

In RA, the heterogeneous DN B cell compartment is expanded and dominated by IgG isotype. TCZ can modulate the mutational status of DN Ig isotype receptors over 1 year. Interestingly, the frequency of DN B cells in RA may serve as a baseline predictor of subsequent EULAR response to TCZ.

**Electronic supplementary material:**

The online version of this article (doi:10.1186/s13075-015-0580-y) contains supplementary material, which is available to authorized users.

## Introduction

Rheumatoid arthritis (RA) is a chronic, systemic, inflammatory, autoimmune disease characterized by inflammation of the joints, which results in their progressive destruction [1,2]. Recent studies suggest that B cells play an important role in the development and progression of RA through several mechanistic pathways, and the subsequent polyclonal activation of B cells [3]. By producing different inflammatory cytokines and autoantibodies, they may directly drive pathologic inflammation [4]. Moreover, memory B cells have gained particular interest in evaluating response during therapies from biologics that have shown promising results in treatment of RA. Different cytokines are involved in B cell differentiation, maintenance and survival. They are distinctly implicated in each phase of the pathogenesis of RA-promoting autoimmunity, maintaining chronic inflammatory synovitis and driving the destruction of joint tissue [5]. Biological agents targeting key proinflammatory cytokines, such as tumor necrosis factor alpha (TNF-α) and interleukin 6 (IL-6) have been substantially advanced in the treatment of autoimmunity [6]. IL-6 is a multifunctional pleiotropic cytokine acting as stimulator of both B and T cell functions. It is produced by various cells of the innate immune system (for example macrophages, dendritic cells, mast cells, neutrophils), B cells, and to some extent by CD4 effector T helper (Th) cells [7]. IL-6 also influences various cell types and has multiple biological activities through its unique receptor system [8]. It has been described as a late-acting B cell differentiation factor that is involved in *in vitro* differentiation of B cells into antibody-forming cells and germinal center reactions. In addition to its involvement in immune responses, it also regulates hematopoiesis, the acute phase response and inflammation. Dysregulation of IL-6 production and its pathological role in different autoimmune diseases have been well documented and highlight IL-6 and its signaling cascade as a potential target for autoimmune therapy [9-13]. Consequently, tocilizumab (TCZ), a humanized anti-IL-6 receptor (IL-6R) monoclonal antibody (mAb) against the alpha chain of IL-6R, which prevents binding of IL-6 to membrane and soluble IL-6R, was developed and has been licensed for the treatment of RA [14]. TCZ has shown convincing clinical efficacy by reduction of signs/symptoms and a marked inhibition of radiological progression [11].

**Figure 1:**
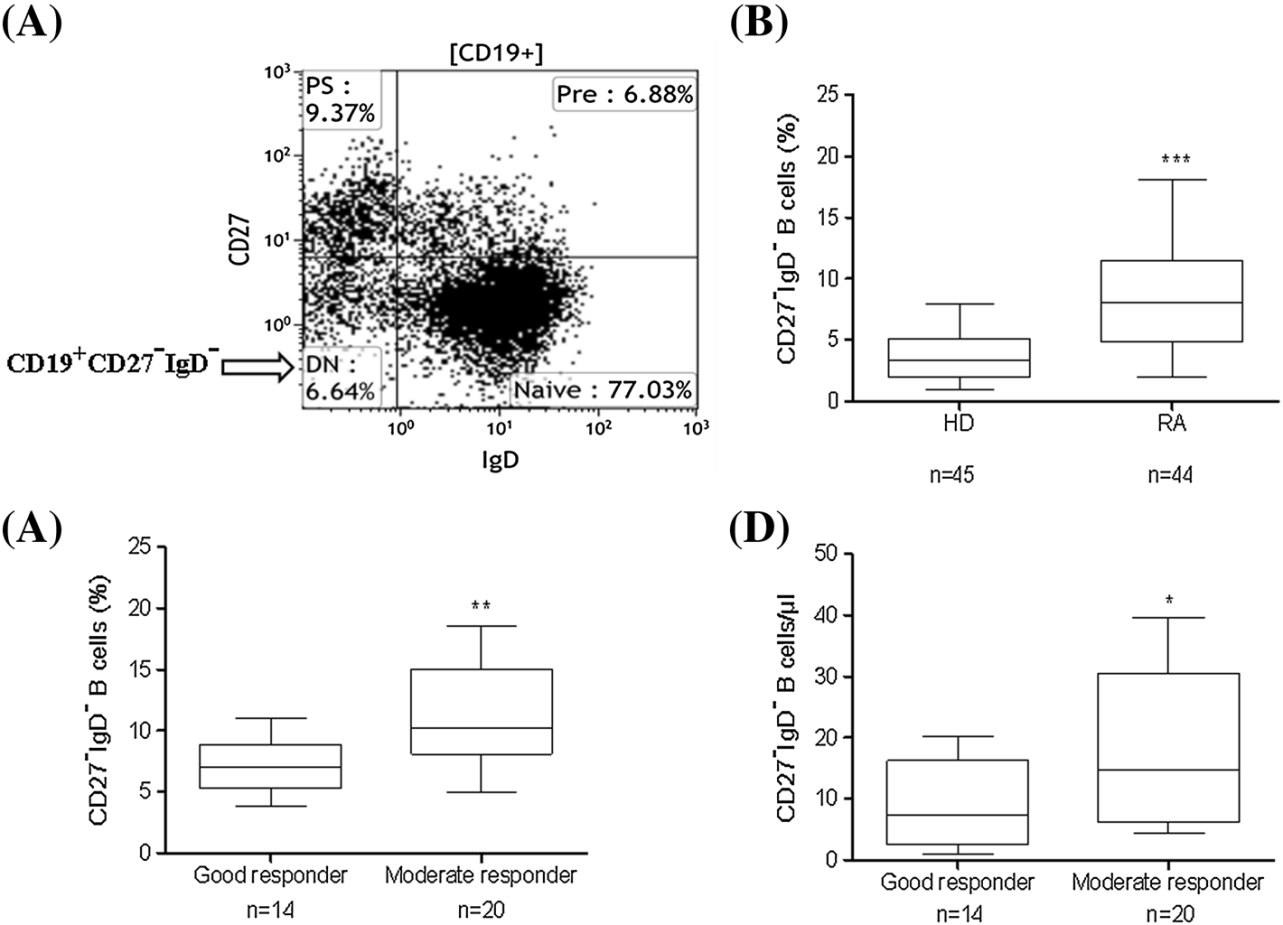
**Phenotype analysis of CD27-IgD- B cells in RA patients and their relation to EULAR response. (A)** Representative FACS plot. Characterization of (CD27-IgD-) DN B cells, PS = post-switch (CD27 + IgD-), Pre = pre-switch (CD27 + IgD+) and naïve (CD27-IgD+) B cells. **(B)** Comparison of DN B cells in RA patients and HD. DN B cells in RA patients (n = 44) and HD (n = 45) show a significantly higher percentage of the frequency of DN B cells in RA patients (*P* <0.0001). **(C)** EULAR response to IL-6R inhibition. Week 12 EULAR good responders (BL DAS28 = 5.1 ± 0.3) to TCZ have significantly (*P* = 0.006) lower frequency of DN B cells at baseline compared to EULAR moderate responders (BL DAS28 = 5.3 ± 0.3). **(D)** EULAR responses to IL-6R inhibition (absolute cell numbers). Week 12 EULAR good responders have significantly (*P* = 0.05) lower absolute DN B cell numbers at baseline compared to moderate responders. Data shown in box-whisker plot where boxes represent 25th to 75th percentiles and the lines within the boxes represent the median. *P* values were determined by Mann-Whitney *U* test using GraphPad Prism 5. (^***^ *P* <0.0001, ^**^ *P* <0.001 and ^*^ *P* <0.05). BL DAS28, baseline disease activity score 28; DN, double-negative; EULAR, European League Against Rheumatism; HD, healthy donor; Ig, immunoglobulin; IL-6R, interleukin-6 receptor; RA, rheumatoid arthritis; TCZ, tocilizumab.

Functionally distinct B cell subsets can be defined by the phenotype expression of CD27 and immunoglobulin D (IgD). Human peripheral memory B cells are mainly discriminated from naïve B cells by the phenotypic expression of CD27 (a member of the tumor necrosis factor receptor (TNFR) family) and presence of somatic hypermutation (SHM) in their Ig variable genes [15,16]. CD27 expression by B cells has been considered a hallmark for SHM and their memory. CD27+ memory B cells are a heterogeneous population comprising of pre-switch (IgD + CD27+) and post-switch (IgD-CD27+) B cell subsets [13,17,18]. There are still unanswered questions about the exact identification of memory B cells based on CD27 expression, since recent studies in these lines have shown a double-negative (DN) population (CD19 + CD27-IgD-) that bears all signatures of memory B cells [19-21] (Figure 1A). A very large portion of DN (CD27-IgD-) B cells express mutated Ig and an evaluation of telomere length, expression of the anti-apoptotic molecule Bcl2, and absence of the ATP-binding cassette B1 transporter (ABCB1) have been used to discriminate them from naïve CD27- B cells and relate them to the memory B cell compartment [22,23]. Even though DN memory B cells mainly express switched Ig isotypes, they have a reduced rate of SHM compared to post-switch B cells. This has been hypothesized to be due to either an impaired germinal center (GC) formation or resembling a distinct lineage of memory B cells [23,24]. In systemic lupus erythematosus (SLE), DN B cells are expanded and could be linked to autoimmunity by analysis of the specific autoantibodies including 9G4 expression [19]. So far, the nature of DN B cells has still not been fully delineated in general as well as in autoimmune diseases.

Our previous studies of memory B cell subsets during *in vivo* IL-6R inhibition indicated phenotypic and molecular changes in pre-and post-switch memory B cells [13,14,25]. In RA, DN B cells have not been thoroughly studied and there is scarce information in the literature. Therefore, we initiated the current study to analyze the DN B cell compartment in RA in more detail by phenotypic and molecular analyses of the different isotypic DN B cell receptors, their immunoglobulin receptor (Ig-R) mutational pattern and their modulation by *in vivo* IL-6R and TNF-α inhibition.

## Methods

### Patients and healthy donors

Peripheral blood was taken from 44 rheumatoid arthritis patients (RA) with a median age of 54 (range 33 to 79) years and 49 healthy age-matched donors (HD) for B cell phenotype and molecular analysis. All patients met the American College of Rheumatology revised criteria for RA [26]. The patients had median disease duration of 9 (range 2 to 33) years and 77% were female. Informed consent was obtained from all patients according to the protocol approved by the ethics committee of the university hospital, Würzburg, Germany. Patients who failed to respond to treatment with standard disease-modifying antirheumatic drugs (DMARDs) including methotrexate (MTX) were eligible and enrolled in the study. A dose of 8 mg/kg TCZ was administered every 4 weeks as a 60-minute infusion in combination with MTX. Alternatively, adalimumab (ADA) (40 mg every 2 weeks) in combination with MTX was given as control. The clinical primary end point was set at 12 weeks, with an extension period up to 24 weeks and follow-up till 1 year. Forty TCZ-treated patients were followed at baseline, weeks 12 and 24 for phenotypic analysis. A total of 33/40 patients were rheumatoid factor (RF)-positive and 28/40 patients were anti-citrullinated protein antibodies (ACPA)-positive. Furthermore, all the ACPA-positive patients were positive for RF, 5/40 ACPA-negative patients were RF-positive and 7/40 patients were negative for both RF and ACPA. Four patients left the study after week 12. For correlation with clinical parameters, 36 patients undergoing TCZ therapy were studied, 14/36 reached a European League Against Rheumatism (EULAR) good response at week 12, 20/36 were EULAR moderate responders and 2/36 were non-responders to TCZ. The baseline disease activity score using 28 joint counts (DAS28) and C-reactive protein (CRP) levels of EULAR good responders (5.1 ± 0.3; 0.9 ± 0.2 mg/dl) and moderate responders (5.3 ± 0.3; 0.8 ± 0.2 mg/dl), respectively were similar [27]. Clinical characteristics of the patients undergoing TCZ therapy are summarized in Table 1. Nine RA patients under TCZ therapy, four patients under anti-TNF-α therapy and three HD were selected for analysis of mutational patterns of Ig-Rs by single-cell polymerase chain reaction (PCR) technique.

**Table 1:** **Characteristics of patients treated with tocilizumab therapy**

	**Baseline (n = 36)**	**Week 12 (n = 36)**	**Week 24 (n = 36)**	**1 year (n = 36)**
Age, median (range) years	54 (33-79)			
% female	77%			
Disease duration, median (range) years	9 (2-33)			
RF positive	36			
ACPA positive	28			
RF/ACPA positive	28			
	mean ± SEM			
DAS28 score	5.2 ± 0.3	3.3 ± 0.3^ψ^	2.7 ± 0.2^ψ^	1.9 ± 0.2^ψ^
CRP, mg/dl	0.9 ± 0.1	0.14 ± 0.06^ψ^	0.09 ± 0.03^ψ^	0.06 ± 0.02^ψ^
ESR, mm/hour	32.1 ± 3.4	9.8 ± 1.6^ψ^	7.2 ± 0.9^ψ^	5.6 ± 0.8^ψ^
Patient’s VAS	58.3 ± 4.4	48.6 ± 4.8^ψ^	34.8 ± 5.4^ψ^	21.5 ± 4.8^ψ^
IgA-RF U/ml	122.3 ± 33.3	99.9 ± 26.3^ψ^	63.3 ± 19.8^ψ^	-
IgA total U/ml	265.4 ± 20.8	237.2 ± 19.5^ψ^	203.1 ± 18.9^ψ^	-

The primary end point of the study was a reduction in the disease activity score in 28 joints (DAS28) at week 12. Except where indicated otherwise, values are the mean ± standard error of the mean (SEM). ^ψ^ *P* <0.05 versus baseline and Mann-Whitney *t* test was used for statistics. RF, rheumatoid factor; ACPA, anti-citrullinated protein antibodies; CRP, C-reactive protein; ESR, erythrocyte sedimentation rate; VAS, visual analog scale (100 mm); IgA, immunoglobulin A.

### Flow cytometric analysis

Whole blood staining was used for phenotype studies and peripheral blood mononuclear cells (PBMCs) were used for single-cell PCR approach. Whole blood and PBMCs were stained with the following monoclonal antibodies: anti-CD45-krome orange (Beckman Coulter (Brea, CA, USA), cat no. 96416), anti-CD14-PC5.5 (Beckman Coulter, cat no. A70204), anti-CD19-APC-Alexa Fluor 750 (Beckman Coulter, cat no. A94681), anti-CD19-APC (BD Pharmingen (San Jose, CA, USA), cat no. 555415), anti-CD27-PE (BD Pharmingen, cat no. 555441), anti-CD27-PC7 (Beckman Coulter, cat no. A54823), anti-CD27-ECD (Beckman Coulter, customized), anti-IgD-FITC (BD Pharmingen, cat no. 555778), anti-IgA-FITC (Beckman Coulter, cat no. 732610), anti-IgG-PECy7 (BD Pharmingen, cat no. 561298) and anti-IgM-APC (BioLegend (San Diego, CA, USA) cat no. 314510). After staining, cells were analyzed by 10-color flow cytometer (Navios, Beckman Coulter). B cells were identified by CD19+ cells gated on CD45 + CD14- lymphocytes in combination with forward scatter versus side scatter gating on CD45+ lymphocytes. B cell subpopulations were identified by using CD27 and IgD expression gated on B cells. Expression of IgG, IgA and IgM was analyzed for CD27 + IgD- post-switch and CD27-IgD- DN B cells, respectively. At least 20,000 CD19+ events were collected for each analysis. The total number of B cells of various phenotypes was calculated per microliter of blood, based on the frequency of these cells among the lymphocytes and the absolute number of white blood cells.

### Single-cell sorting

PBMCs were isolated by Ficoll-Paque Plus separation (Pharmacia Biotech, Freiburg, Germany) using the standard protocol. Single B cell sorting from PBMCs was carried out as described previously [28]. Briefly, B cells were stained for CD19-APC, CD27-PE and IgD-FITC and gated by forward/side scatter; doublets for CD19 positivity were excluded. DN B cells were defined as CD19 + IgD-CD27-. Individual DN B cells were sorted in a 96-well plate containing lysis buffer by using FACS ARIA-III cell sorter (Becton Dickinson, San Jose, CA, USA). Lysis buffer was comprised of Triton X-100, bovine serum albumin (BSA), oligo (dT)_15_ primer, dithiothreitol, RNasin, and RNAse-free double-distilled H_2_O or RNAse-free H_2_O.

### cDNA preparation and nested PCR

A mixture comprising RT-PCR buffer, reverse transcriptase and nucleotides from Titan One Tube RT-PCR System (Roche Diagnostics, Mannheim, Germany) was added into a 96-well plate. cDNA synthesis was carried out at 50°C for 1 hour. V_H_3 gene rearrangements comprising the largest V_H_ family were amplified by nested PCRs using family-specific primers as previously described [29]. Briefly, the cycle program consisted of one cycle at 95°C for 5 min, 50 to 58°C for 1 min, 72°C for 1 min, followed by 30 cycles of 94°C for 1 min, 50 to 58°C for 30 s, 72°C for 1 min, followed by 5 min incubation at 72°C. Using 5 μl of the PCR product from first amplification as template, the second round of nested PCR was conducted with a primer specific. In order to differentiate different isotypes in DN B cells, C_H_1μ-, C_H_1α- and C_H_1γ-specific PCRs were carried out using cycle program as described previously [30]. The error rate due to the *Taq* polymerase used in the amplification process was estimated to be 1 × 10^-4^ mutations/bp [31]. V_H_ family-specific PCR products (350 bp) were separated via electrophoresis using 1.5% agarose gel and further purified by using MinElute Gel Extraction kit (Qiagen, Hilden, Germany) according to the manufacturer’s instructions.

### Sequencing and analysis

Gel extracted PCR products of Ig-V_H_3 and isotype-specific Ig were amplified using BigDye Terminator Cycle Sequencing Ready Reaction kit followed by sequencing in genetic analyzer ABI PRISM 310 (Applied Biosystems, Carlsbad, CA, USA). A total of 2,407 sequences were analyzed by matching their closest germline counterparts using the online program JOINSOLVER [32].

### Statistical analysis

Statistical analysis was performed using GraphPad Prism 5.0 (GraphPad Software, San Diego, CA, USA) and SPSS Statistics 22.0 (IBM Corp., Armonk, NY, USA). Values were always compared with baseline levels by using the nonparametric Wilcoxon matched pair test and Mann-Whitney *U* test. Univariate logistic regression was used to calculate odd ratios and correlation using Pearson r. The values ≤0.05 were considered to be significant. ^***^P <0.0001, ^**^P <0.001 and ^*^P <0.01.

## Results

### Double-negative (CD19 + IgD-CD27-) B cells are expanded in RA

Based on surface expression of IgD and CD27 (Figure 1A), human peripheral CD19 + B cells were divided into four subsets: mature naïve B cells (IgD + CD27-), pre-switch memory B cells (IgD + CD27+), post-switch memory B cells (IgD-CD27+) and DN B cells (IgD-CD27-). Frequencies of peripheral DN B cells were determined in RA patients (n = 44; median age approximately 54 years) and healthy donors (n = 45; median age approximately 52 years). We found significantly enhanced frequencies of DN B cells in RA patients compared to HD (Figure 1B). Here, RA patients had a median (range) of 8.4 (2.0 to 18.1) percent of CD19+ DN B cells as compared with 3.3 (1.0 to 7.9) percent in HD. Notably, absolute numbers of DN B cells of RA with 10.7 (1.9 to 32.9) cells/μl were comparable to HD with 9.6 (1.7 to 29.5) cells/μl.

### DN B cells correlate to clinical response to tocilizumab

During TCZ therapy, DAS28 of all patients declined significantly from 5.2 at baseline (BL) to 3.3, 2.7 and 1.9 at week 12, 24 and 1 year (*P* <0.0001). The inflammatory parameters erythrocyte sedimentation rate (ESR) and CRP were also significantly reduced (Table 1). The clinical response was maintained up to 1 year and there were no serious adverse events or infections during the study. At week 12, during IL-6 inhibition with TCZ therapy 14 out of 36 patients reached a EULAR good response, 20 patients reached a moderate response and 2 patients were non-responders. Interestingly, we found that good EULAR responders to TCZ at week 12 had a significantly lower frequency of DN B cells compared to moderate responders, at BL. The same correlation was also found for absolute numbers of DN B cells at BL (Figure 1C-D). There was no significant correlation between frequency of DN B cells with DAS28 (R = -0.007; *P =* 0.97) and CRP levels (R = 0.07; *P =* 0.70). Univariate logistic regression analysis revealed that the frequency of DN B cells at BL is inversely correlated with a subsequent EULAR good response with a significant (*P* = 0.024) odds ratio of 1.48 (95% confidence interval (CI) as 1.05 to 2.06) favoring EULAR good response. This indicates that the percentage of DN B cells is elevated in RA and correlated with EULAR response to TCZ.

### Phenotypic isotype distribution of CD27-IgD- DN and post-switch B cells in RA and their modulation during IL-6R inhibition

**Figure 2:**
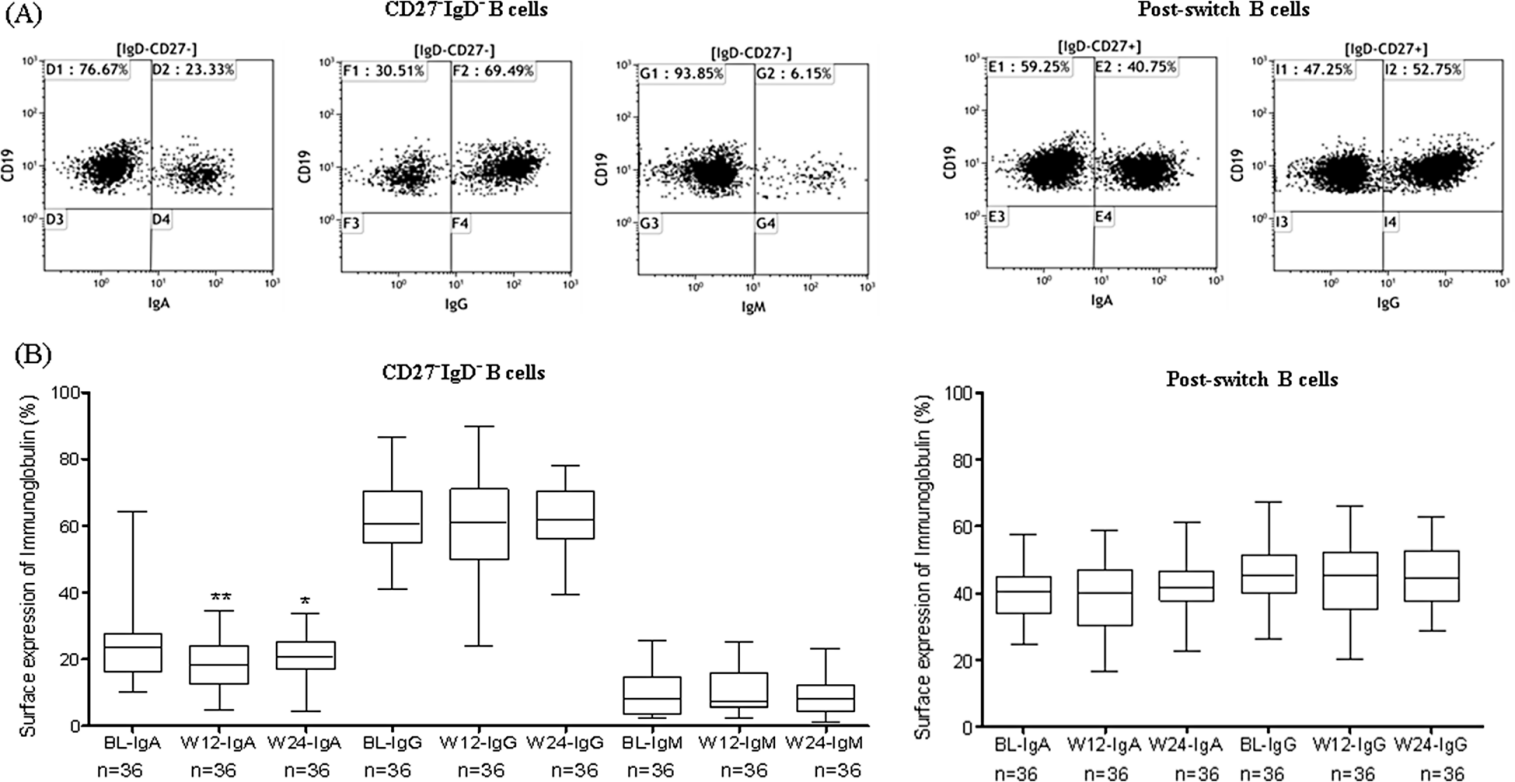
**Surface expression of immunoglobulin isotypes during IL-6R inhibition. (A)** Representative FACS plots showing the expression of immunoglobulin isotypes, IgA, IgG and IgM on gated DN and post-switch B cells. **(B)** During IL-6R inhibition, IgA+ DN B cells decreased significantly from 24.7 (10.0 to 64.2) percent median (range) to 18.4 (4.8 to 34.7) at week 12 (*P* = 0.004) and 20.5 (4.6 to 33.8) at week 24 (*P* = 0.04) respectively. There were no remarkable changes in relative IgG+ and IgM+ DN B Cells. **(C)** IgA+ and IgG+ post-switch B cells were not influenced during IL-6R inhibition. BL, baseline, W12, week 12 and W24, week 24. DN, double-negative; Ig, immunoglobulin; IL-6R, interleukin-6 receptor.

In order to reveal the isotypic distribution of DN B cells, we measured the surface expression of IgG, IgA and IgM isotypes and followed them in RA patients (n = 36) during TCZ treatment till week 24. DN B cells showed a heterogeneous mixture of cells containing IgG+, IgA+ and IgM+ expressing B cells with a dominance of IgG isotype. In detail, DN B cells contained a median (range) of 60.6% (40.9 to 86.7) IgG+ isotype of total DN B cells, 24.7% (10.0 to 64.2) of IgA+, and 8.0% (2.3 to 25.8) of IgM+ B cells. For comparison, post-switch memory B cells displayed an almost equal distribution of IgG+ and IgA+ cells; 45.3% (26.2 to 67.3) IgG+ and 42.7% (24.5 to 57.4) IgA+ (Figure 2). The distribution of IgG+, IgA+ and IgM+ DN B cells in HD were comparable to RA patients (Additional file 1). Since IL-6 inhibition has been shown to influence B cell maturation, we studied DN B cells during TCZ therapy. TCZ did not change the elevated frequency of the overall population of CD27-IgD- DN B cells. However, by analyzing the Ig isotypes in CD27-IgD- DN B cells we found a significant relative decrease of IgA+ DN cells from 24.7% (10.0 to 64.2) median (range) to 18.4% (4.8 to 34.7) at week 12 (*P* = 0.004) and 20.5% (4.6 to 33.8) at week 24 (*P* = 0.04), respectively. We did not find any remarkable changes in IgG+ or IgM+ B cells (Figure 2B). Moreover IgA+ DN B cells declined also significantly in absolute numbers (*P* <0.05) Additional file 2A). In contrast, the frequency of both IgA+ and IgG+ in the post-switch B cells compartment was not influenced during TCZ therapy (Figure 2C), indicating a more dynamic IgA response in the DN compartment. Interestingly serum IgA levels as well as RF-IgA both declined significantly during TCZ therapy (Table 1).

### Somatically mutated Ig-receptors of DN B cells and their modulation during IL-6R

In a subsequent study we performed molecular analysis of DN B cells and analyzed in detail the Ig-R of individually sorted cells from HDs (n = 3) and from RA patients during TCZ (n = 9) or anti-TNF-α therapy (n = 4). As shown previously, [33] Ig-V_H_3 gene rearrangements of DN B cells Ig-R showed less mutation compared to pre-switch and post-switch B cells. Specifically, mutational frequency of DN B cells was 4.0 ± 0.2% compared to 4.5 ± 0.2% for pre-switch B cells and 6.2 ± 0.3% for post-switch B cells. Moreover, the mutational frequency of DN B cells was comparable in RA and HD (Additional file 2A).

**Figure 3:**
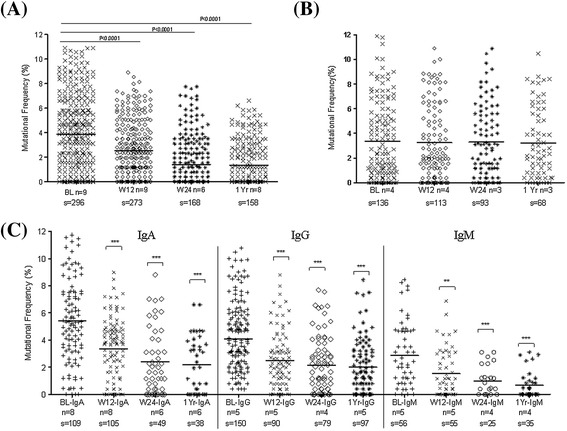
**Ig-receptor somatic hypermutation of gene rearrangements during IL-6R and TNF-α inhibition in DN B cells. (A)** Ig-V_H_3 gene rearrangements during IL-6R inhibition. Reduction in mutational frequency of Ig-V_H_3 gene rearrangements of DN B cells from the peripheral blood of RA patients during TCZ therapy. The mutational frequency was significantly reduced at week 12, 24 and 1 year (^***^ *P* <0.0001 compared to BL). **(B)** Ig-receptor somatic hypermutation of V_H_3 gene rearrangements during TNF-α inhibition. During TNF-α inhibition by adalimumab, a comparable mutational frequency of V_H_ gene rearrangements of DN B cells from the peripheral blood of RA patients were observed at all time points. **(C)** Ig-receptor somatic hypermutation of isotype-specific IgA+, IgG+ and IgM+ gene rearrangements during IL-6R inhibition in DN B cells. At the BL, the mutational frequency of IgA+ DN B cells is significantly higher compared to IgG+ and IgM+ DN B cells. During TCZ therapy, all isotypes IgA+, IgG+ and IgM+ DN B cells showed a significantly reduced mutational frequency (^***^ *P* <0.0001,^**^ *P* <0.001). In a scatter plot, the line represents mean of all values and each dot depicts the mutational frequency of a single sequence. *P* values were determined by Wilcoxon test using GraphPad Prism 5. (BL, baseline; W12, week 12; W24, week 24; n, number of individuals; s, number of sequence analyzed). DN, double-negative; HD, healthy donor; Ig, immunoglobulin; IL-6R, interleukin-6 receptor; RA, rheumatoid arthritis; TCZ, tocilizumab; TNF-α, tumor necrosis factor alpha.

During TCZ therapy however, the mutational frequency of Ig-R of DN B cells was significantly reduced at week 12, week 24 and 1 year (Figure 3A). The mutational frequency of DN B cells decreased from 4.04 ± 0.2% (BL) to 2.52 ± 0.2% at week 12 (*P* <0.0001), 1.98 ± 0.3% at week 24 (*P* <0.0001) and 1.89 ± 0.3% (*P* <0.0001) at 1 year respectively during TCZ (Figure 3A). Consistent with that, highly mutated sequences (>20 mutations per sequence) decreased from 46.2 ± 5.1% (BL) to 28.7 ± 5.5% at week 12 (*P* = 0.018), 23.1 ± 6.8% at week 24 (*P* = 0.035) and 17.3 ± 3.9% at 1 year (*P* = 0.0003) during TCZ therapy. In parallel, a significant increase of unmutated (0 mutation per sequence) sequences from 15.1 ± 3.4% (BL) to 22.7 ± 3.7% at week 12 (*P* = 0.05), 32.1 ± 4.6% at week 24 (*P* = 0.005) and 37.1 ± 4.6% at 1 year (*P* = 0.004) was found (data not shown). In order to evaluate TCZ-specific influences on Ig gene rearrangements, we also studied patients (n = 4) who underwent TNF-α inhibition using adalimumab therapy (Figure 3B). Mutational frequency of Ig-R did not change in DN B cells during adalimumab therapy (Figure 3B). Both therapeutic anti-cytokine interventions significantly reduced clinical activity. During TCZ, DAS28 was reduced from a mean of 5.2 at baseline to 3.3 at week 12, 2.7 at week 24 and 1.9 at 1 year. During anti-TNF-α therapy DAS28 declined from a mean of 4.7 at BL to 3.1 at week 12, 2.7 at week 24 and 1.6 at 1 year. These results indicate that anti-TNF-α therapy does not influence the process of somatic hypermutation of DN B cells as is observed during IL-6R inhibition.

### Ig-specific isotypes are modulated during IL-6R inhibition

Since DN B cells are a heterogeneous population of IgG, IgA and IgM isotypes, we addressed if the TCZ therapy has distinct influences on different isotypes. We performed isotype-specific PCRs from single-sorted DN B cells, and amplified the C_H_1μ-, C_H_1γ- and C_H_1α-specific immunoglobulin gene rearrangements (Figure 3C). At baseline, the isotype-specific Ig-R analysis revealed a significantly higher mutational frequency of IgA+ DN B cells (*P* = 0.01) compared to IgG+ DN B cells at BL. As expected IgM+ DN B cells showed the lowest mutational frequency. However during TCZ therapy, all three isotypes (IgA+, IgG+ and IgM+) of DN B cells showed a significantly reduced mutational frequency. Mutational frequency of IgA+ cells were found to be reduced (mean ± standard error of the mean (SEM)) from 5.42 ± 0.30% (BL) to 3.35 ± 0.22% at week 12 (*P* <0.0001), 2.41 ± 0.34% at week 24 (*P* <0.0001) and 2.26 ± 0.33% (*P* <0.0001) at 1 year, respectively. The mutational frequency of IgG+ cells reduced from 4.45 ± 0.20% (BL) to 2.51 ± 0.22% at week 12 (*P* <0.0001), to 2.16 ± 0.23% at week 24 (*P* <0.0001) and 2.00 ± 0.20% (*P* <0.0001) at 1 year, respectively. Similarly, the mutational frequency of IgM+ cells also decreased from 2.87 ± 0.32% (BL) to 1.55 ± 0.23% at week 12 (*P* = 0.0016), 0.98 ± 0.21% at week 24 (*P* = 0.0008) and 0.67 ± 0.17% (*P* <0.0001) during 1-year TCZ treatment (Figure 3C). These data are consistent with the overall V_H_3 analysis of individual DN B cells during TCZ treatment (Figure 3A).

### CDR3 length and targeting of mutational hotspots motifs during IL-6R inhibition

**Figure 4:**
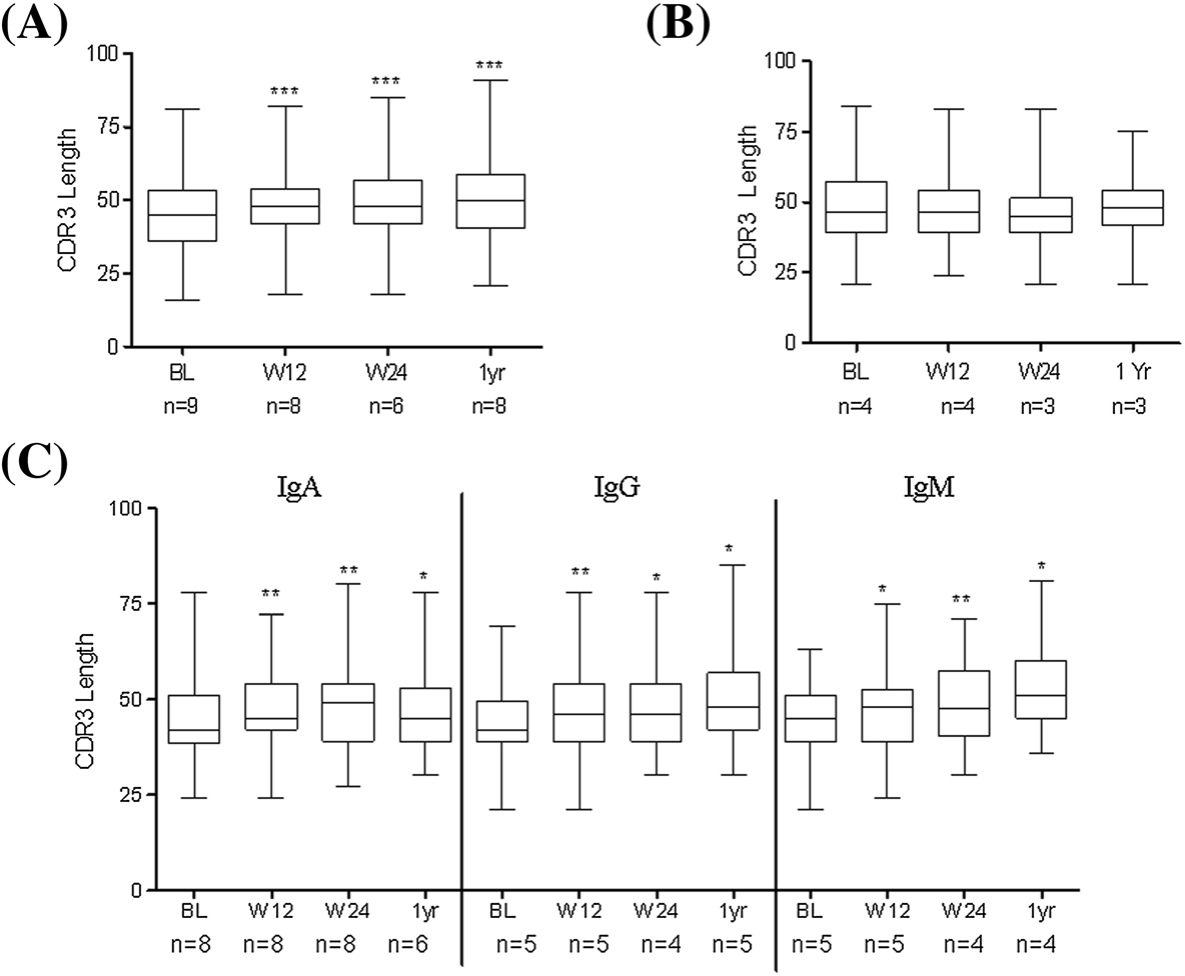
**CDR3 length during IL-6R and TNF-α inhibition. (A)** Significant increase in the CDR3 length of Ig-V_H_ gene rearrangements of DN B cells from the peripheral blood of RA patients during IL-6R inhibition. (^***^ *P* <0.0001). **(B)** During TNF-α inhibition, a comparable CDR3 length of Ig-V_H_ gene rearrangements of DN B cells were observed at all time points. **(C)** There is significant increase in the length of the CDR3 in all three isotypes specific to DN B cells during IL-6R inhibition. *P* values were determined by Wilcoxon test using GraphPad Prism 5. CDR3, the length of the third complementary determining region; DN, double negative; IL-6R, interleukin-6 receptor; RA, rheumatoid arthritis; TNF-α, tumor necrosis factor alpha.

**Figure 5:**
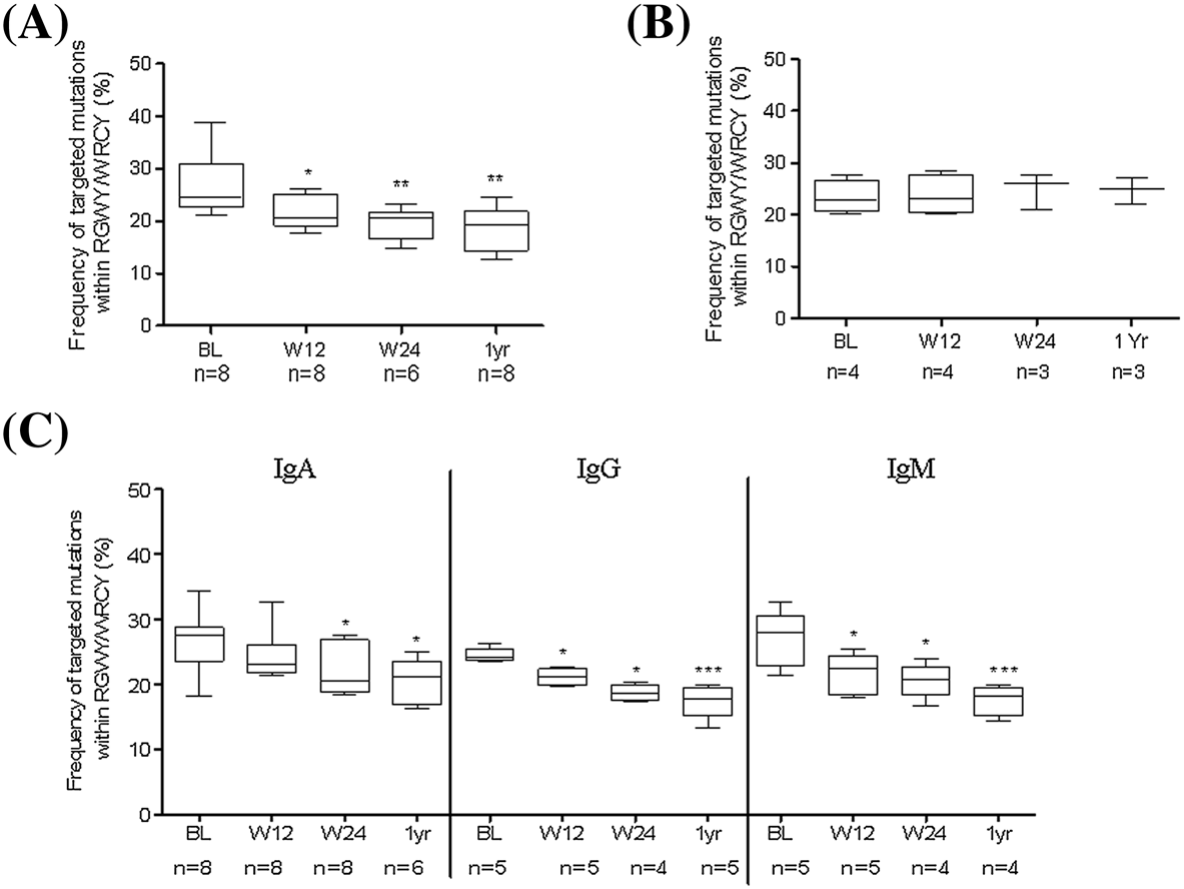
**Frequency of targeted mutations within hotspot motifs during IL-6R and TNF-α inhibition. (A)** A significant reduction in frequency of targeted mutations within RGWY/WRCY is found in Ig-V_H_ gene rearrangements of DN B cells during TCZ therapy (^*^ *P* = 0.046, ^**^ *P* = 0.004). **(B)** During TNF-α inhibition, a comparable mutational hotspot targeting of V_H_ gene rearrangements of DN B cells were observed at all time points. **(C)** A significant reduction in frequency of targeted mutations within RGWY/WRCY is found in all three isotypes specific to DN B cells during IL-6R inhibition. *P* values were determined by Wilcoxon test using GraphPad Prism 5. DN, double negative; Ig, immunoglobulin; IL-6R, interleukin-6 receptor; TCZ, tocilizumab; TNF-α, tumor necrosis factor alpha.

Along with SHM, the length of the third complementary determining region (CDR3) is considered a signature of antigen contact and T cell help, and it represents imprints of selection. CDR3 lengths of V_H_ gene rearrangements at different time points are shown in Figure 4A. We showed that CDR3 length of DN B cells increased from median (range) 45.0 (16.0 to 81.0) base pair (bp) (BL) to 48.0 (18.0 to 85.0) bp at week 12 (*P* = 0.0004), 48.0 (18.0 to 85.0) bp at week 24 (*P* <0.0001) and 50 (21.0 to 91.0) bp (*P* <0.0001) at 1 year during TCZ therapy (Figure 4A). Analysis of the frequency of targeted RGYW/WRCY mutations (R, purine; Y, pyrimidine; W, A/T) showed that it decreased significantly from median (range) 24.6 (21.1 to 38.9) at baseline to 20.5 (17.7 to 26.1) at week 12 (*P* = 0.046), 20.7 (14.9 to 23.4) at week 24 (*P* = 0.004) and 19.2 (12.6 to 24.5) at 1 year (*P* = 0.004) during TCZ therapy (Figure 5A). Frequency analysis of mutational hotspot targeting and CDR3 length analysis during anti-TNF-α therapy also revealed no changes in DN B cells Ig-receptor (Figures 4B and 5B). Furthermore, the frequency analysis of targeted mutations revealed that there was a significant decrease of this targeted mutations from baseline to week 12, 24 and 1 year in all isotype-specific IgA+, IgG+ and IgM+ DN B cells during TCZ therapy (Figure 5C). Likewise, CDR3 length of isotype-specific DN B cells increased from BL to week 12, week 24 and 1 year during TCZ therapy (Figure 4C). Overall, these findings indicate that TCZ therapy not only modulates acquired SHMs in Ig-R of DN B cells, but also exerts effects on the length of CDR3.

## Discussion

With increased understanding of mechanistic pathways of B cell involvement in autoimmune diseases, a number of studies have provided evidence that B cells play a central role in the pathogenesis of RA and that certain subsets may be exploitable as biomarkers. Memory B cells, in particular, seem to be important in driving chronic inflammation [28,34]. Targeting pre-switch and post-switch memory B cells has been found to be important for the response to rituximab [35]. However the impact of different memory B cell subsets has not been thoroughly studied. We recently showed that *in vivo* IL-6R inhibition by TCZ does influence the peripheral pre-switch and post-switch memory B cells in RA [14,25]. Because CD27-IgD- DN B cells also carry features of memory B cells [19,20], we were particularly interested in studying their behavior in active RA, since there is scant literature available for RA. DN B cells confer a notable component of the peripheral blood B cell compartment in RA patients (Figure 1A). Similar to others [35,36], we found a relatively higher frequency of the DN B cell subset in RA patients compared to healthy donors, whereas the absolute numbers of DN B cells were comparable to HD is mainly due to lymphopenia in RA patients. Elevated DN memory B cells have been described to be related to aging, persistent antigen stimulation and seem to reflect overall B-cell hyperactivity [19,23,37-39]. In our study, elevation of DN B cells did not reflect simply inflammation since their number did not correlate to CRP or DAS28 levels.

We describe a heterogeneous DN B cell population containing IgG+, IgA+ and IgM+ DN B cells with pre-dominance of the IgG clearly different from post-switch memory B cells (Figure 2A). During TCZ therapy, there was moderate influence on the phenotypic composition of the DN compartment. Nevertheless, the IgA-DN B cell phenotype seems to be particularly amenable to IL-6R inhibition - the percentage of IgA+ DN B cells significantly decreased during TCZ therapy (Figure 2B). Also absolute numbers of IgA+ DN cells were reduced significantly (Additional file 3). Moreover we see a significant reduction of serum IgA (Table 1 and reference [14]) as well as RF-IgA levels (Table 1). This highlights the IL-6 susceptibility of the IgA isotype. However, it should be pointed out that our data provides no direct relation of IgA-DN B cells to declining IgA serum factors. Nevertheless IL-6 has been ascribed a critical player in the development of local IgA antibody responses and a remarkable reduction of mucosal IgA-producing cells has been reported in mice with targeted disruption of the gene that encodes IL-6 [40]. Future experiments need to address how peripheral IgA+ DN B cells may be related to the mucosal immune system.

SHM of immunoglobulin gene rearrangements is a hallmark of B cell maturation into memory cells after antigen encounter. Despite their mainly switched phenotype, we also found the mutational frequency of DN B cells in RA to be significantly lower compared to post-switch B cells and similar to pre-switch B cells. This has been previously reported for other diseases and older people [19,20,24,25]. We found a significant decrease in mutational frequency of the B cell receptor (BCR) at week 12, 24 and over a year during TCZ therapy (Figure 3A). TCZ treatment again induces profound changes with reduced mutational status in all three isotypes (Figure 3C). Interestingly, IgA+ DN B cells also harbored the highest mutational frequency compared to IgG+ and IgM+ DN B cells, which was evident in RA as well as healthy donors (Additional file 2B).

It is known that SHM favors defined mutational hotspots. In particular, RGYW/WRCY motif mutations are preferentially targeted by T cell-dependent signals including CD40-CD40 ligand interactions [41]. The decrease in frequency of targeted mutations within RGYW/WRCY and increase in CDR3 during IL-6R inhibition indicates that during therapy the DN B cell population consists of less antigen-experienced B cells. This effect seems mainly influenced by IL-6R inhibition therapy since we did not observe any change in these parameters during anti-TNF-α therapy using adalimumab (Figure 3B), although both biologics resulted in good reduction of clinical inflammation. In a previous study looking at the pre-switch B cell compartment, we observed a similar pattern with adalimumab failing to influence Ig-R mutation [25]. Therefore, it seems likely that IL-6R blockade does affect B cell maturation *in vivo* to a substantial extent. However, at the moment it is not clear how direct and indirect effects on B cells contribute to the observed effects since TCZ is also reported to reduce activated CD4+ T cells as early as 12 weeks after treatment [42]. Data still leaves these questions open. DN B cells are hypothesized to resemble transient effector B cells [43]. A recent report suggested that Syk++ B cells lacking CD27 expression represent a unique atypical memory-like B cell, which may be relevant for IgG+ plasmablast generation [44]. Furthermore they might derive from incomplete GC or alternatively from extrafollicular reactions [43]. Since mutational frequencies of DN and pre-switch B cells are significantly lower than typical post-switch B cells, it might be suggested that either of these cells are pre-germinal centre or leave the GCs quickly by shedding CD27. On the other hand, DN B cells still could fail to upregulate CD27 expression before leaving GCs since CD27-negative memory B cells have also been found and isolated from human tonsils [45]. Since shedding or downregulation of CD27 have been reported as an indication of continuous antigen stimulation and T cell exhaustion, our observed connection of DN B cells numbers to response to IL-6R inhibition may reflect a state of chronic B cell hyperactivity closely linked to IL-6 [37-39]. Furthermore, the data are consistent with the idea that DN memory B cells are distinctly generated or have a shorter half-life than conventional post-switch memory B cells resulting in a measurable impact of IL-6R blockade on DN B cells.

The search for predictive biomarkers to individualize treatment strategies in RA is of current interest. For IL-6R inhibition with TCZ, predictive markers for response have not yet been identified. In our study, we were able to relate the level of peripheral DN B cells to the clinical response to TCZ (Figure 1B-C). Patients achieving EULAR good response at week 12 had significantly lower percentages and absolute numbers of DN B cells at baseline before TCZ therapy. In univariate logistic regression analysis, a lower frequency of DN B cells showed an odds ratio of 1.48 for achieving a EULAR good response to TCZ. It is important to note that the frequency of DN B cells is not correlated to CRP or DAS28 levels at baseline. Thus CD27-IgD- DN B cells may indicate an elevated B cell activity in RA that is not reflected by these inflammatory markers but is susceptible to TCZ. This may allow future studies to exploit DN B cells as biomarkers for response to TCZ.

## Conclusions

In summary, we demonstrate a significantly higher population of CD27-IgD- DN B cells in RA patients. These DN B cells are a mixture of somatically mutated IgG, IgA and IgM isotype-bearing cells with a dominance of IgG isotype. TCZ therapy results in a decrease in the frequency of IgA+ DN B cells in particular. In parallel, a decrease of serum IgA and RF-IgA levels is observed. On the molecular level, TCZ therapy reduces the mutational frequency and RGYW hotspot targeting in all DN B cells over a period of 1 year. Based on these current findings, DN memory B cells may serve as a candidate biomarker for response to TCZ therapy as lower baseline values of these cells were related to higher proportions of EULAR good responders.

## Additional files

Additional file 1:
**Surface expression of DN B cells immunoglobulin isotypes in HD and RA patients.** **(A)** The distribution of IgA+, IgG+ and IgM+ DN B cells were comparable in HD and RA patients before undergoing therapy. **(B)** Similarly in post-switch memory B cells, distributions of IgA+ and IgG+ cells were comparable. Additional file 2:
**Ig-receptor somatic hypermutation of V**_**H**_ **3 and isotype-specific IgA+, IgG+ and IgM+ gene rearrangements of DN B cells in HD and RA.** **(A)** Comparable mutational frequency of V_H_3 gene rearrangements of DN B cells from the peripheral blood of RA patients before undergoing therapy and HD. **(B)** Isotype specific IgA+, IgG+ and IgM+ gene rearrangements of DN B cells were comparable. Additional file 3:
**Surface expression of immunoglobulin isotypes during IL-6R inhibition (absolute cell numbers).** **(A)** IgA+ DN B cell absolute numbers per μl are decreased significantly from median (range) 1.9 (0.42 to 9.5) to 1.4 (0.4 to 6.0) at week 24 (*P* = 0.04). Absolute cell numbers of IgG+ and IgM+ DN B cells are showing a trend for reduced numbers during TCZ therapy. **(B)** Absolute cell numbers of isotype specific IgG+ and IgA+ post-switch B cells shows a weak trend to reduced numbers during TCZ therapy. Total number of patients = 36. BL = baseline, W12 = week 12 and W24 = week 24. *P* values were determined by Mann-Whitney *U* test using GraphPad Prism 5. (^***^ *P* <0.0001, ^**^ *P* <0.001 and ^*^ *P* <0.05).

## Abbreviations

ABCB1: ATP-binding cassette B1 transporter
ACPA-positive: anti-citrullinated protein antibodies
ADA: adalimumab
BL: baseline
bp: base pair
BSA: bovine serum albumin
BCR: B cell receptor
CDR3: length of the third complementary determining region
CRP: C-reactive protein
DAS28: disease activity score using 28 joint counts
DMARDs: disease-modifying antirheumatic drugs
DN: double negative
ESR: erythrocyte sedimentation rate
EULAR: The European League Against Rheumatism
GC: germinal center
HD: healthy donor
Ig-R: immunoglobulin receptor
IgD: immunoglobulin D
IL-6R: interleukin-6 receptor
mAb: monoclonal antibody
MTX: methotrexate
PBMCs: peripheral blood mononuclear cells
PCR: polymerase chain reaction
RA: rheumatoid arthritis
RF: rheumatoid factor
SHM: somatic hypermutation
SLE: systemic lupus erythematosus
TCZ: tocilizumab
Th: T helper
TNF-α: tumor necrosis factor alpha
TNFR: tumor necrosis factor receptor
