# Supplementary figures and images for: CD27-IgD- memory B cells are modulated by in vivo interleukin-6 receptor (IL-6R) blockade in rheumatoid arthritis
Source: Arthritis Res Ther. 2015 Mar 14;17(1):61. doi: 10.1186/s13075-015-0580-y (PMC4415279; doi:10.1186/s13075-015-0580-y)

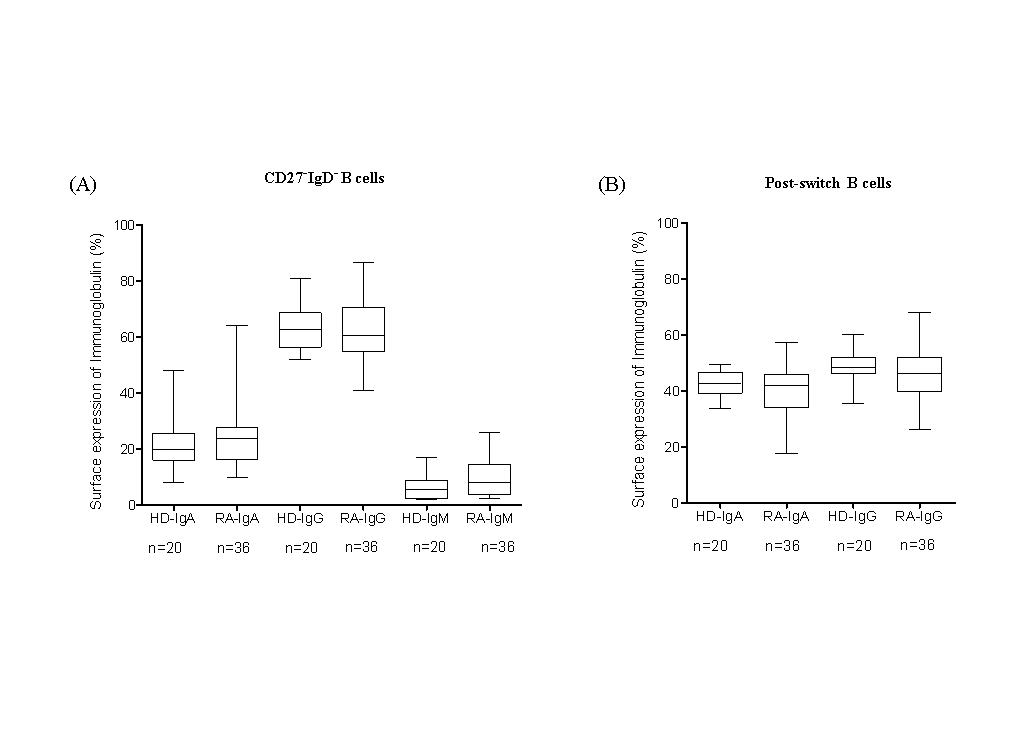

Supplement: Additional file 1: — Surface expression of DN B cells immunoglobulin isotypes in HD and RA patients. (A) The distribution of IgA+, IgG+ and IgM+ DN B cells were comparable in HD and RA patients before undergoing therapy. (B) Similarly in post-switch memory B cells, distributions of IgA+ and IgG+ cells were comparable. [file 13075_2015_580_MOESM1_ESM.tiff]

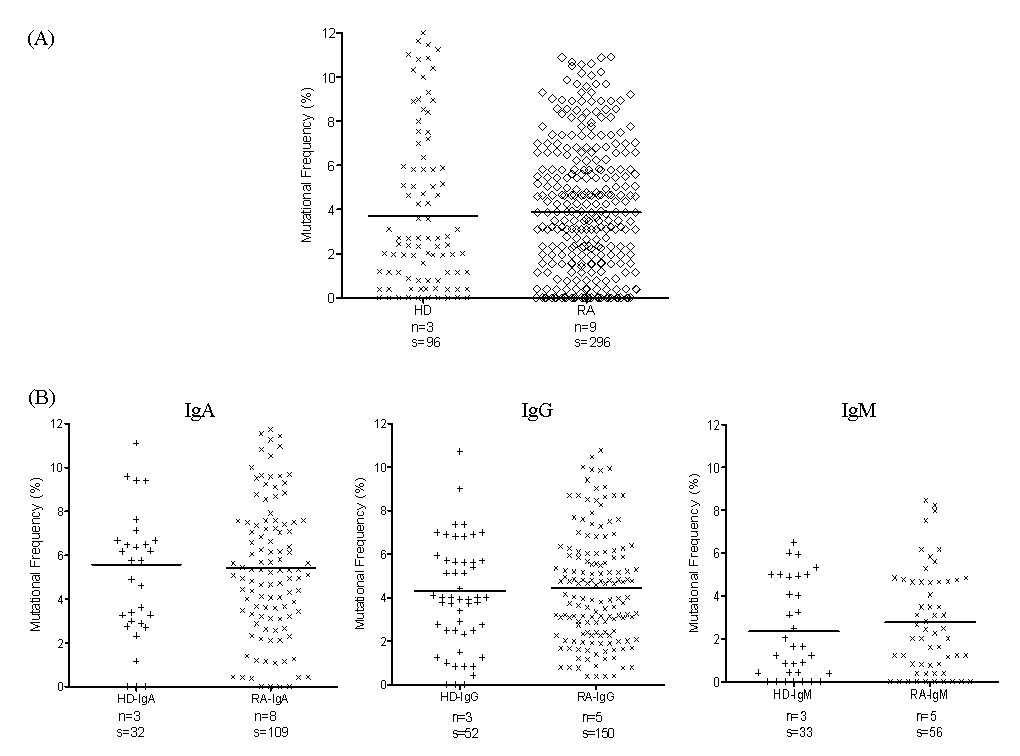

Supplement: Additional file 2: — Ig-receptor somatic hypermutation of V H 3 and isotype-specific IgA+, IgG+ and IgM+ gene rearrangements of DN B cells in HD and RA. (A) Comparable mutational frequency of VH3 gene rearrangements of DN B cells from the peripheral blood of RA patients before undergoing therapy and HD. (B) Isotype specific IgA+, IgG+ and IgM+ gene rearrangements of DN B cells were comparable. [file 13075_2015_580_MOESM2_ESM.tiff]

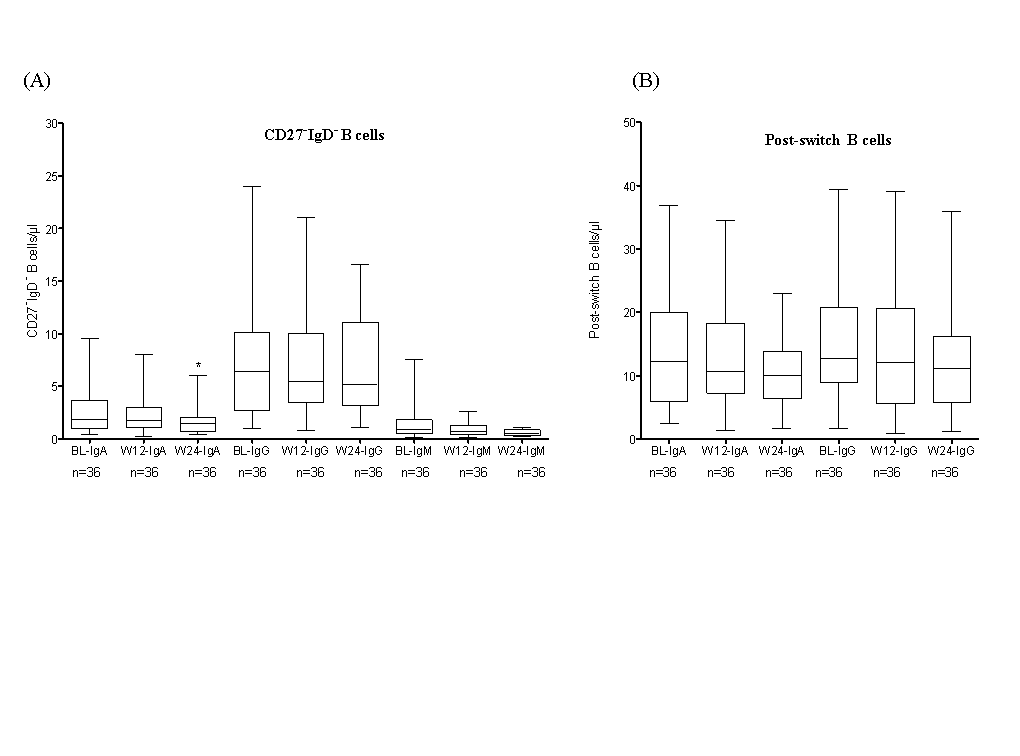

Supplement: Additional file 3: — Surface expression of immunoglobulin isotypes during IL-6R inhibition (absolute cell numbers). (A) IgA+ DN B cell absolute numbers per μl are decreased significantly from median (range) 1.9 (0.42 to 9.5) to 1.4 (0.4 to 6.0) at week 24 (P = 0.04). Absolute cell numbers of IgG+ and IgM+ DN B cells are showing a trend for reduced numbers during TCZ therapy. (B) Absolute cell numbers of isotype specific IgG+ and IgA+ post-switch B cells shows a weak trend to reduced numbers during TCZ therapy. Total number of patients = 36. BL = baseline, W12 = week 12 and W24 = week 24. P values were determined by Mann-Whitney U test using GraphPad Prism 5. (*** P <0.0001, ** P <0.001 and * P <0.05). [file 13075_2015_580_MOESM3_ESM.tiff]
